# Supplementary material for: Content-rich biological network constructed by mining PubMed abstracts
Source: BMC Bioinformatics. 2004 Oct 8;5:147. doi: 10.1186/1471-2105-5-147 (PMC528731; doi:10.1186/1471-2105-5-147)
Supplement: Additional File 5 — The original Chilibot query results of the term "long-term potentiation (LTP)" and 22 other terms, limiting the latest references analyzed to the years 1990, 1995, 2000, and 2004. [file 1471-2105-5-147-S5.bz2 › chilibotAdditionalFile5/ltp2004/html/AMPA_SYNAPTOPHYSIN.html]

 


 **AMPA** and **SYNAPTOPHYSIN** 
  
Found 29 abstracts in PubMed,  **29 abstracts were retrieved and analyzed**.  


---

 Search Google  |
 PDF files only 
|  EDU domain only 

---

**Interactive relationship** (e.g. stimulation, inhibition, etc)

**Parallel relationship** (e.g. studied together, co-existance, homology, etc.)

- Using these antibodies, in conjunction with GluR2 specific and  **synaptophysin**  antibodies, we have directly localized and quantified surface expressed native  **AMPA**  and NMDA receptors on cultured living hippocampal neurons during development.  Ref: 11050112 J Neurosci, 2000
- In parallel, there was a substantial increase in the expression levels of the presynaptic markers  **synaptophysin** , synaptotagmin, and synapsin I and of GluR1  **AMPA**  receptor subunits.  Ref: 12595243 Mol Cell Neurosci, 2003
- The distributions of the presynaptic marker  **synaptophysin** , the  **AMPA**  type glutamate receptor subunit GluR1, and the putative NMDA receptor clustering protein PSD 95 were not affected by blockade.  Ref: 9354327 Neuron, 1997
- Long term maintenance was evident for other synapse related proteins, including  **synaptophysin** , neural cell adhesion molecule isoforms, and an  **AMPA**  receptor related antigen, as well as for certain structural and cytoskeletal components.  Ref: 8773255 Hippocampus, 1995
- Since the nerve terminal markers  **synaptophysin**  and the SV2 glycoprotein were not detectably different in the two groups of mice, the age related reduction in  **AMPA**  receptors is not likely to be due to a general decrease in synaptic density.  Ref: 1327416 Brain Res, 1992
- The time course of the known stripping of synapses from the cell somas of axotomized motoneurons was studied by using  **synaptophysin**  antibodies and compared with  **AMPA**  and mGluR1a receptor changes.  Ref: 10982465 J Comp Neurol, 2000
